# Increased incidence of adult-onset Still’s disease in association with COVID-19 vaccination and SARS-CoV-2 infection

**DOI:** 10.1186/s13023-023-02651-3

**Published:** 2023-03-10

**Authors:** Maxime N. Gottschalk, Max Heiland, Susanne Nahles, Robert Preissner, William A. Petri, Stephanie Wendy, Saskia Preissner

**Affiliations:** 1 Department of Oral and Maxillofacial Surgery, Charité – Universitätsmedizin Berlin, Corporate Member of Freie Universität, Berlin, Humboldt-Universität zu Berlin, Berlin Institute of Health, Augustenburger Platz 1, 13353 Berlin, Germany; 2 Institute of Physiology and Science-IT, Charité – Universitätsmedizin Berlin, Corporate Member of Freie Universität Berlin, Humboldt-Universität zu Berlin, Berlin Institute of Health, Philippstr. 12, 10115 Berlin, Germany; 3 Departments of Medicine, Pathology, Microbiology, Immunology and Cancer Biology, University of Virginia, Charlottesville, VA 22908-1340 USA

**Keywords:** SARS-CoV-2, COVID-19, Vaccination, Adult-onset, Morbus Still, Rare disease

## Abstract

**Background:**

Adult-onset Still’s disease (AOSD) is a multi-system, auto-inflammatory disease characterized by fever, arthralgia, typical rash, leukocytosis, sore throat, and liver dysfunction, among other symptoms. Retrospective studies about the frequencies of AOSD have shown that this disease is very rare. However, there has been an increased scientific interest in the last 2 years, as numerous case studies on AOSD have been published. These case studies describe the occurrence of AOSD after SARS-CoV-2 infection and/or COVID-19 vaccination.

**Methods:**

We analyzed the incidence of AOSD to examine a potential association between AOSD and SARS-CoV-2 infection and/or COVID-19 vaccination. The TriNetX dataset consists of 90 million patients. We found 8474 AOSD cases, which we analyzed regarding SARS-CoV-2 infection and/or vaccination status. We also analyzed the cohorts considering demographic data, lab values, co-diagnoses and treatment pathways.

**Results:**

We divided the AOSD cases into four cohorts: primary cohort (AOSD), Cov cohort (AOSD + SARS-CoV-2 infection), Vac cohort (AOSD + COVID-19 vaccination) and Vac + Cov cohort (AOSD + COVID-19 vaccination + SARS-CoV-2 infection). For the primary cohort, we found an annual incidence of 0.35 per 100.000. We found an association between AOSD and SARS-CoV-2 infection and/or COVID-19 vaccination. According to the numerical analysis, the incidence of AOSD doubled for the Cov cohort and Vac cohort. Moreover, the incidence of AOSD was 4.82 times higher for Vac + Cov cohort. The lab values for inflammatory markers were increased. Co-diagnoses such as rash, sore throat, and fever appeared in all AOSD cohorts, with the highest occurrences in the AOSD + COVID-19 vaccination + SARS-CoV-2 infection cohort. We identified several lines of treatments, mainly in association with adrenal corticosteroids.

**Conclusions:**

This research supports the assumption of an association between AOSD and SARS-CoV-2 infection and/or COVID-19 vaccination. However, AOSD remains a rare disease and the usage of vaccines to fight the COVID-19 pandemic should not be questioned because of the increased incidence of AOSD.

**Supplementary Information:**

The online version contains supplementary material available at 10.1186/s13023-023-02651-3.

## Background

Still’s disease was first mentioned by G. Still in 1897 as juvenile chronic polyarthritis in 22 children [1]. It had long been accepted that these forms of arthritis are only diagnosed in children, but almost 75 years later Eric Bywaters described the first 14 cases of the Still’s disease syndrome in female patients between 17 and 35 years with the same symptoms. His report indicated that Still’s disease is not an age-related version of polyarthritis. Moreover Bywaters, argued for a nosological differentiation [1]. Nonetheless, recent data have revealed that adult-onset Still’s disease (AOSD) is a rare, multi-system, auto-inflammatory disease [2] characterized by fever, arthralgia, rash, leukocytosis, sore throat, lymphadenopathy and/or splenomegaly, liver dysfunction, and the absence of rheumatoid factor and antinuclear antibodies [3]. As these characteristics are also caused by other conditions, the diagnosis of AOSD is partially made by excluding other conditions [4]. However, a few attempts have been made to define diagnostic criteria. The criteria of Yamaguchi et al. have the highest sensitivity [4].

There is a lack of epidemiological data for AOSD [5]. Based on a couple of regional studies and case series, there are estimations regarding the (annual) incidence of the disease. There are limitations to these studies: They are retrospective and the number of patients and the examined incidence vary significantly (Table 1).

**Table 1:** Data about the frequency of adult-onset Still’s disease (AOSD) in different countries

Country	Type of study	Number of cases	(Annual) incidence per 100,000 people	Publication	Mean age (years)	Year of the study
Turkey (Thrace region)	Retrospective	42	0.62	[6]	44.5	2003–2014
Northern Norway	Retrospective	13	0.4	[7]	33.8	1990–2000
France	Retrospective	62	0.16	[8]	36	1982–1991

AOSD occurs irrespective of ethnicity and sex; furthermore, no familial accumulation has been reported [9]. So far, neither a genetic nor another triggering factor has been identified for the manifestation of AOSD [10]. It is known that interleukin (IL)-1 has a major role in in the pathogenesis [11]. According to Giampietro et al. [12], one of the major events in the pathogenesis is the increased release of active IL-1ß.

There have been a few case studies describing the diagnosis AOSD after COVID-19 vaccination and/or SARS-CoV-2 infection using the Yamaguchi classification (Table 2).

**Table 2:** Cases of adult-onset Still’s disease (AOSD) after SARS-CoV-2 infection and/or COVID-19 vaccination

Sex	Age (years)	Occurrence of symptoms	Publication	Criteria used for diagnosis	Type of vaccination
Female	29	6 months after the diagnosis of SARS-CoV-2 infection	[11]	Yamaguchi classification	–
Male	27	Recovered from SARS-CoV-2- infection 8 weeks prior	[13]	Yamaguchi classification	–
Male	36	1 day after first COVID-19 vaccination	[14]	Yamaguchi classification	ChAdOx1 nCoV-19
Male	43	10 days after receiving the second dose of the vaccination	[15]	Yamaguchi classification	BNT162b2 mRNA
Female	56	7 days after receiving the second vaccination	[15]	Yamaguchi classification	BNT162b2 mRNA
Male	53	8 weeks after COVID-19 vaccination	[16]	Yamaguchi classification	ChAdOx1 nCoV-19/AZD1222
Female	45	5 days after the second dose of the COVID-19 vaccination	[17]	Yamaguchi classification	mRNA-1273 COVID-19 vaccine
Female	36	10 days after the first dose of vaccination	[18]	Yamaguchi classification	BNT162b2 mRNA

Most of these cases occurred after vaccination [14, 18]. The authors did not mention sex-related differences. The reported temporal association between the initial onset of AOSD and SARS-CoV-2 infection and/or COVID-19 in the published case reports strengthens the assumption of a causal connection, but numerical considerations are missing. Hence, we evaluated the association between the risk of AOSD and SARS-CoV-2 infection and/or COVID-19 vaccination and the possible patient-related parameters in a larger multi-center cohort.

## Methods

The TriNetX Global Health Research Network contains real-world data of about 250 million individuals from over 120 health care organizations (HCOs) across 19 countries. We accessed the database on April 6, 2022 (Additional file 1: Fig. S5). We created four cohorts: For the first cohort (in the following: primary cohort) we set AOSD (ICD-10 Code M06.1) as the inclusion criterion, while the exclusion criteria were SARS-CoV-2 infection and/or COVID-19 vaccination. For the second cohort (Cov cohort), we set SARS-CoV-2 infection followed by AOSD as the inclusion criteria, while the third cohort (Vac cohort) includes COVID-19-vaccinated patients who were diagnosed with AOSD after vaccination. The 4th cohort (Vac + Cov cohort) includes COVID-19-vaccinated individuals, infected with SARS-CoV-2 after vaccination and then diagnosed with AOSD. The incidence was calculated for 20 years per 100,000 patients (the AOSD frequency per 100,000 people was divided by 20). We adapted our numbers to annual incidence for better comparability to the data cited in the introduction. In addition, we analyzed demographic data, lab values and co-diagnoses. Treatment pathways have been analyzed on September 22, 2022.

For two cohorts, namely Vac + Cov and Cov cohort we performed 1:1 propensity score matching for age and sex. After defining the primary outcome as “AOSD” (ICD-10 Code M06.1), risk ratio (RR) and odds ratio (OR) were calculated. Statistical analysis was performed using the Log-Rank test, whereby *p* 0.05 was defined as significance threshold.

## Results

We identified 8474 AOSD cases. There are 6847 patients in the group with AOSD but without SARS-CoV-2 infection or COVID-19 vaccination before onset. Our analysis revealed an annual incidence of 0.35 per 100,000 people in this group. The incidence of AOSD is higher in the Cov cohort, Vac cohort and Vac + Cov cohort (Table 3). The Cov cohort includes 1521 AOSD cases, with an incidence of 0.75 per 100,000. The incidence for the Cov cohort is double that of the primary cohort. The Vac cohort has a similar incidence: 205 AOSD cases for an incidence of 0.81 per 100,000. With 1.69 per 100,000 Vac + Cov cohort shows the highest incidence of AOSD. The incidence for this cohort is 4.82 times higher than the incidence for the primary cohort. After propensity score matching for age and sex for Vac + Cov cohort and Cov cohort, RR and OR were determined as 1.71 (95% CI confidence interval upper = 2.44 and lower = 1.2). The elevated risk for the Vac + Cov cohort to develop AOSD is statistically significant (*p* = 0.0024; Log-Rank test).

**Table 3:** The incidence of adult-onset Still’s disease (AOSD) alone or after SARS-CoV-2 infection and/or COVID-19 vaccination

Disease	Cohort	Total number of patients w/o consideration of AOSD	Number of cases	Age (years), mean ± standard deviation	Sex		Annual incidence (per 100,000 people)
					Female (%)	Male (%)	
AOSD	Primary cohort	93,499,559	6487	57 ± 20	69	31	0.35
AOSD + SARS-CoV-2 infection	Cov cohort	10,103,789	1521	59 ± 18	71	29	0.75
AOSD + COVID-19 vaccination	Vac cohort	1,266,336	205	64 ± 14	63	37	0.81
AOSD + COVID-19 vaccination + SARS-CoV-2 infection	Vac + Cov cohort	771,711	261	63 ± 15	72	28	1.69

All cohorts have a female predominance regarding the incidence of AOSD and SARS-CoV-2 infection and/or a COVID-19 vaccination. Demographic details for the different cohorts and a modified CONSORT flow chart can be found in die Additional file 1: Figs. S1–S5.

We also found similarities in the cohorts regarding the lab values of C-reactive protein (CRP), ferritin, and the erythrocyte sedimentation rate (ESR), and co-diagnoses (Table 4). All cohorts showed increased CRP values (normal value < 5 mg/L). While the CRP value for the primary, Cov and Vac + Cov cohorts are comparable, the CRP value of the Vac cohort is much lower. The ferritin levels increased markedly in all cohorts (normal ranges 20–250 ng/mL). The values are comparable for the primary cohort as well as for Cov cohort and Vac + Cov cohorts, but similarly to the CRP values, Vac cohort has much lower ferritin values than the other cohorts. The ESR values of all cohorts increased (normal value < 15–20 mm/h); Vac + Cov cohort has the highest values.

**Table 4:** Lab values and co-diagnoses (fever, rash, sore throat) for the different cohorts

Unit Cohort	Cohort	CRP	Ferritin	ESR	Fever R50	Rash R21	Sore throat R07
		[mg/L], mean ± SD	[ng/mL], mean ± SD	[mm/h], mean ± SD	[%]		
AOSD	Primary cohort	24.1 ± 49	927 ± 4328	27.7 ± 27.7	31	29	37
AOSD + SARS-CoV-2 infection	Cov cohort	23 ± 46.7	846 ± 4082	28.8 ± 27.9	46	43	57
AOSD + COVID-19 vaccination	Vac cohort	13.3 ± 30.1	540 ± 1760	21.4 ± 22.6	27	30	41
AOSD + COVID-19 vaccination + SARS-CoV-2 infection	Vac + Cov cohort	22.3 ± 52.6	862 ± 5531	28.3 ± 26.3	51	45	62

Considering all the cases, the patients mostly experienced a sore throat as a co-diagnosis. Fever and rash were diagnosed less often, with rash being reported the least among co-diagnoses. The Vac + Cov cohort has the highest presence of all three co-diagnoses followed by Cov cohort, which also shows a comparatively high percentage of co-diagnoses. The percentage of co-diagnoses within the primary cohort and Vac cohort are comparatively lower. Rash and sore throat were reported more within Vac cohort than in primary cohort, while fever was reported slightly more within the primary cohort than in the Vac cohort.

AOSD, adult-onset Still’s disease; CRP, C-reactive protein; ESR, erythrocyte sedimentation rate; SD, standard deviation.

**Fig. 1:**
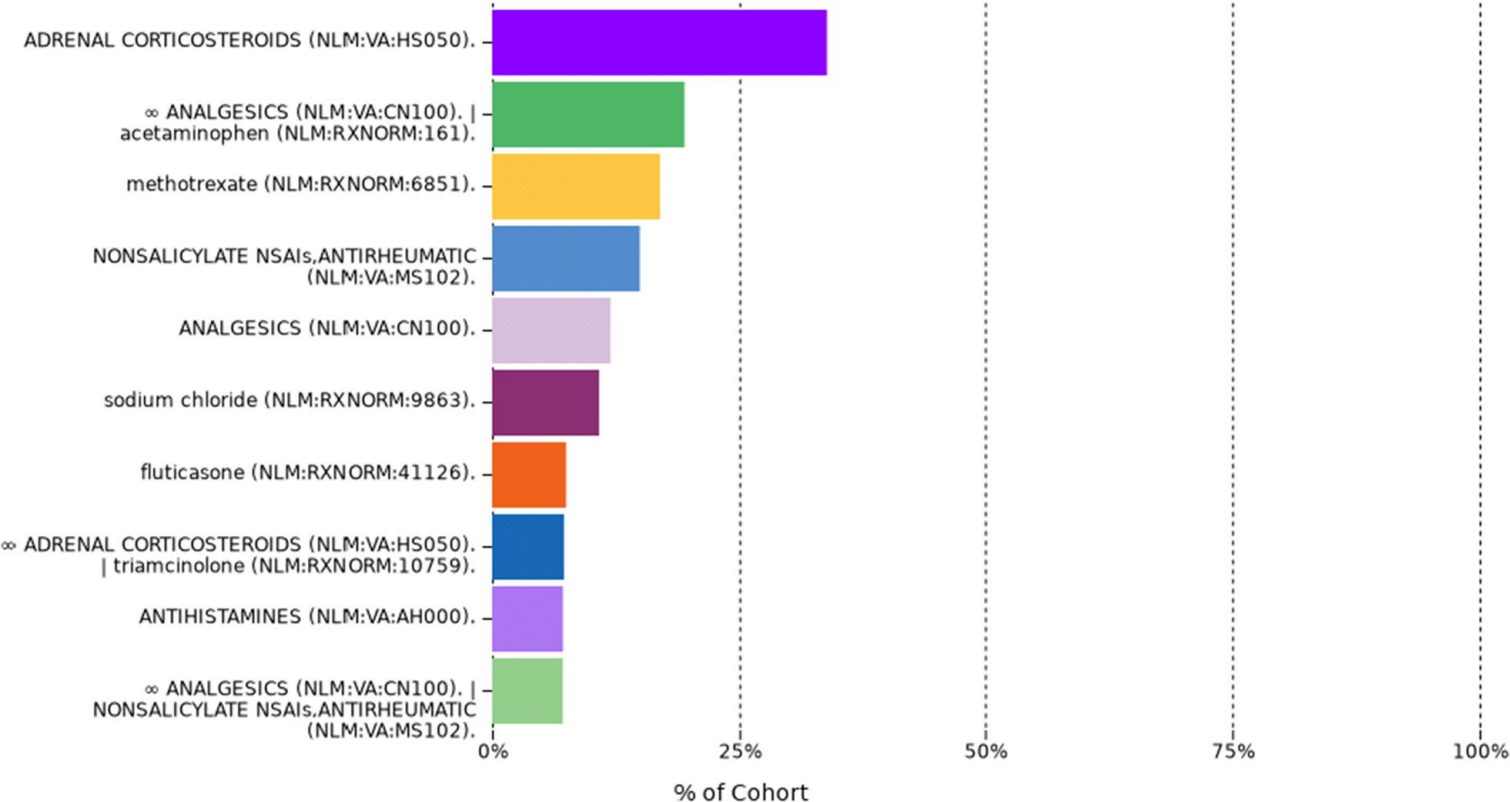
Treatments on pathway: Most common drugs prescribed to patients with AOSD

The analysis of AOSD treatment showed that a majority of patients was treated with adrenal corticosteroids (> 25%). A similar amount of cases was treated with analgesics and acetaminophen or either methotrexate or NSAIs (non-steroidal antirheumatics). Other treatments were analgesics or sodium chloride or antihistamines (see Fig. 1).

**Fig. 2:**
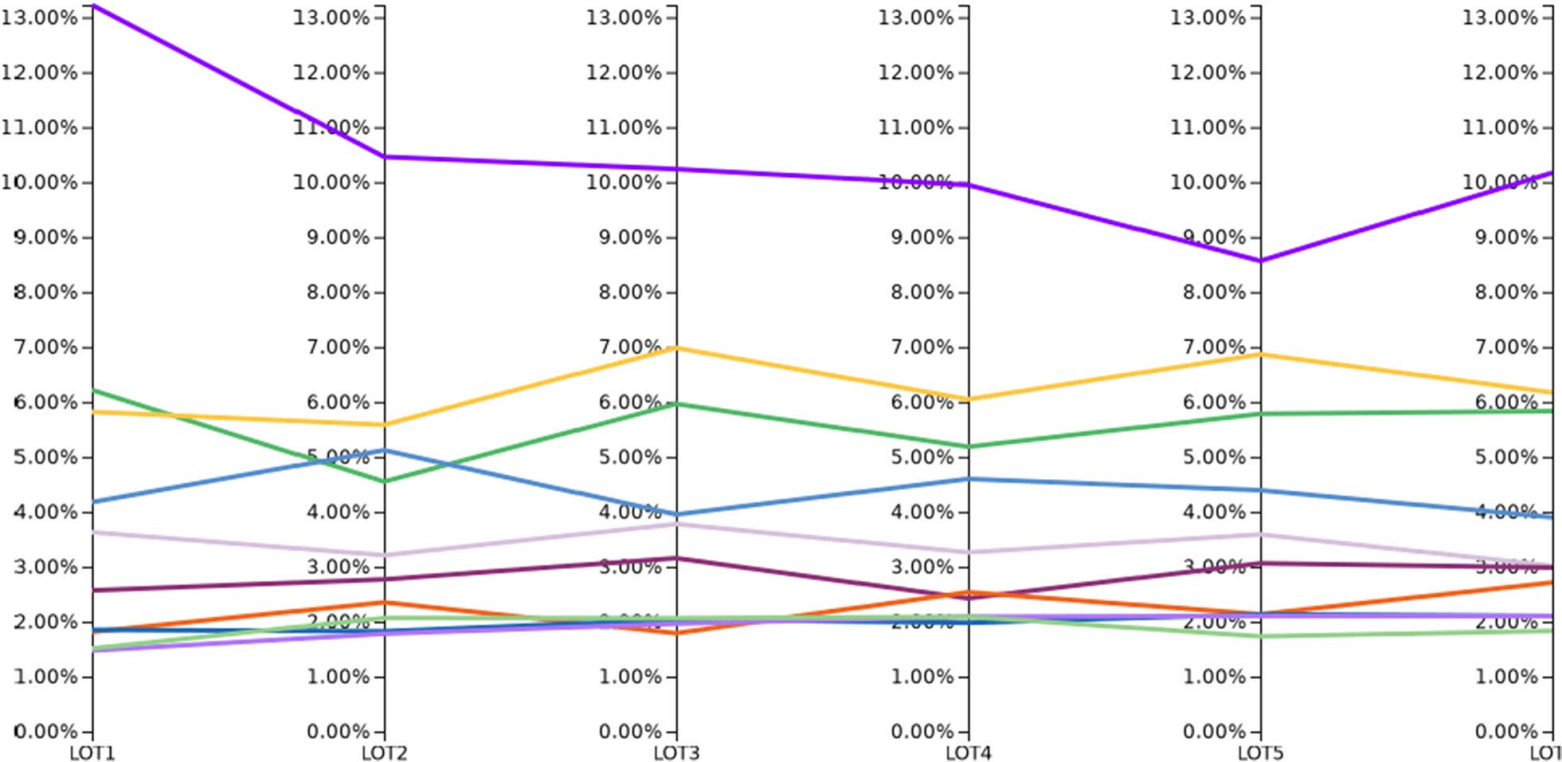
Distribution of treatments within six months (LOT 1–6), drugs see Fig. 1. A line includes any treatment taken within 1 days

The above mentioned treatments were used for up to six months. When looking at the lines of treatment for the first six months after the index event, the treatment does not really vary within the first six months. Solely the treatment with adrenal corticosteroids vary within the first 6 months but adrenal corticosteroids remain the mainly used therapy (Fig. 2). However, within the first six weeks after the onset of AOSD, the therapy with adrenal corticosteroids is reduced by 3–4%.

## Discussion

The incidences in this analysis are higher than the incidences found in the literature. Furthermore, the predominance of the female sex as well as of the Caucasian ethnicity in this analysis are inconsistent with the literature. Additionally, the mean age in our analysis is higher compared with the literature (see Table 1). However, the findings are worth considering because we analyzed a much larger dataset than found in the literature.

Considering the above-mentioned case studies, as well as the two-fold higher incidence for Cov cohort compared with the primary cohort, there seems to be a causal relationship between AOSD and SARS-CoV-2 infection. Leone et al. [14] described the symptoms of AOSD as including systemic inflammation, unremitting fever, high serum ferritin, and a hyperinflammatory syndrome with major organ involvement similar to a severe SARS-CoV-2 infection. Therefore, it may be assumed that both diseases are triggered by the same mechanisms [14]. Regarding therapy, studies have shown that treating AOSD with anti-IL-1 agents is effective [19] and recently published studies have shown positive results after IL-1 therapy of SARS-CoV-2 infection induced hyperinflammation [11]. These findings suggest that there is an association between SARS-CoV-2 infection and AOSD that should be addressed. It is likely that the onset of AOSD after SARS-CoV-2 infection is caused by a misdirected immune response against SARS-CoV-2 [11].

The two-fold higher incidence of AOSD in Vac cohort compared with the primary cohort supports the assumption of a connection between AOSD and COVID-19 vaccination. Even though it is not sufficiently clear how vaccines could influence AOSD [20], some authors have described several assumptions regarding the causes. For example, there are a few cases in which AOSD occurred after the patients had received other vaccines, such as for influenza [21, 22]. The authors assumed that viral proteins from vaccines activate the immune response by molecular mimicry or bystander activation [21]. In addition, there are numerous reports on the side effects of COVID-19 vaccinations. Bindoli et al. [23] reported several cases of hyper-inflammation after COVID-19 vaccination (ChAdOx1 and mRNA). They argue that in genetically predisposed patients, the vaccines cause a pro-inflammatory immune response including excessive production of pro-inflammatory cytokines [23]. In addition, they also observed major improvements after the treatment with anti-IL-1 drugs [23]. Therefore, similarities to the reaction after SARS-CoV-2 infection seem obvious. Compared with the other cohorts, the lab values indicate that the inflammatory reaction within Vac cohort is lower than the other cohorts.

The Vac + Cov cohort has the highest incidence of AOSD. Additional research has to be conducted regarding the hyper-inflammatory reaction after COVID-19 vaccination and SARS-CoV-2 infection. We speculate that the immune response regarding the SARS-CoV-2 infection is even stronger due to prior COVID-19 vaccination. Based on the literature, the second dose of the COVID-19 vaccine could lead to severe AOSD flares due to the stronger immune response to the second dose [20]. Furthermore, booster vaccinations seem to cause an enhanced immune response compared with primary vaccination [24]. SARS-CoV-2 infection as well as COVID-19 vaccination seem to trigger similar reactions. The above-mentioned literature findings may be transferred to the cases of COVID-19 vaccination followed by SARS-CoV-2 infection (instead of a booster vaccination). The Vac + Cov cohort had the most co-diagnoses (fever, rash, and sore throat) of all the cohorts and very high lab values. These findings also support the assumption, that COVID-19 vaccination and a subsequent SARS-Cov-2 infection stimulate the innate immune system even more strongly than in the cases of either SARS-CoV-2 infection or COVID-19 vaccination alone. A control cohort with acute upper respiratory infection (ICD-10 Code J06) was created resulting in an incidence of AOSD similar to our vac cohort. It is likely that misdirected immune responses against acute upper respiratory infections can be a reason for the increased incidence within this control cohort.

It is important to note, that our study has some limitations. It must be mentioned that COVID is a quite recent development, therefore it could be argued that the period of 20 years is not the most suitable time frame for this work. However, we decided for this period to improve the comparability of the found incidences with the incidences from the literature. Nevertheless, a similar consideration within “COVID years” only would also be very beneficial to evaluate the incidence development of AOSD. Especially because of the absence of individual data, it is not possible to assess single cases and a long-term reassessment of the diagnosis according to the individual clinical development (follow-up etc.) is not possible. Therefore, the overall dataset might include cases, which were diagnosed with AOSD at first, however when looking on the long-term development, these cases turn out to be temporary effects. To address this limitation, we conducted an analysis of the distribution of treatments within six months. For about 86% of the patients treatment information was available. The consideration of the (only slightly changing) treatment development within 6 months, supports the assumption that the diagnosis were not temporary effects. Nonetheless there is a high number of cases, which either didn’t obtain a treatment or more likely where the treatment has not been entered into the database. It is therefore important to note, that this study relies on the data quality and the data scope of the contributing HCOs. In this context, the usage of the ICD-10 codes, incorrect coding as well as a missing ICD-10 code for relapses influence the dataset. It is likely that the dataset includes patients who were already diagnosed with AOSD beforehand and who had a relapse of AOSD after COVID-19 and/or COVID-19 vaccine. Therefore, the above-mentioned factors did not trigger a first onset; however, they still had an impact on the occurrence of AOSD. Furthermore, the time of the first onset and the time of coding might be significantly different.

Another reason for missing data within this study is the transition of the patient to a smaller outpatient healthcare facility, which is not using TriNetX. For these cases, it is not possible to evaluate a long-term development. Therefore, this aspect is as a very important limitation to this study. Transitions within the HCOs also influence the analyzed data.

False positive and false negative COVID tests as well as SARS-COV2 infected patient without symptoms may also affect the dataset and the overall share of the different cohorts.

Moreover, missing specification of the COVID-19 subtypes and the different vaccines have an impact on the results of the study as well. We saw that the incidences increase due to Sars-COV2 infection or/and COVID-19 vaccine; however further analyses are necessary to identify whether certain subtypes or a certain vaccine or other infections type lead to an increased onset of AOSD.

Due to the fact that the TriNetX database obtains its data from HCOs from only 19 countries, our findings should be interpreted cautiously regarding the impact on the broad range of worldwide population. However as stated in the introduction, AOSD occurs irrespective of ethnicity, therefore our study remains helpful in order to understand the recent AOSD developments.

## Conclusions

Based on our literature review and considering the recently reported incidences of AOSD, SARS-CoV-2 infection and/or COVID-19 vaccination may trigger the onset of AOSD. However, additional research is necessary to confirm this assumption. As the identified cases share common etiological aspects, exome sequencing would help to identify the cause(s) of AOSD. Moreover, it might be helpful to conduct follow-up studies to identify and analyze AOSD relapses, among other aspects of the disease. The case studies have reported a strong temporal relationship, but it might be possible that the onset of AOSD after SARS-CoV-2 infection and/or COVID-19 vaccination is coincidental. Nevertheless, the higher incidence of AOSD in Cov cohort, Vac cohort and Vac + Cov cohort compared with the primary cohort strengthens the assumption of a relationship. Even though the incidence of AOSD increased significantly after SARS-CoV-2 infection and/or COVID-19 vaccination, AOSD remains an orphan disease. It is important to note that we are not criticizing the usage of vaccines to fight the COVID-19 pandemic. We only want to draw attention to AOSD as a side effect of the vaccine as well as a related disease after SARS-CoV-2 infection, in order to diagnose AOSD and to initiate the right treatment as early as possible.

## Supplementary Information

**Additional file1**. **Figure S1**: Demographics for Primary cohort (AOSD). **Figure S2**: Demographics for Cov cohort (AOSD + SARS-CoV-2 infection). **Figure S3**: Demographics for Vac cohort (AOSD + COVID-19 vaccination). **Figure S4**: Demographics for Vac+Cov cohort (AOSD + COVID-19 vaccination + SARS-CoV-2 infection).** Figure S5**: Modified CONSORT flow chart for the different cohorts.

## Data Availability

The datasets generated and analyzed during the current study are available upon request.
