# Supplementary material for: Increased incidence of adult-onset Still’s disease in association with COVID-19 vaccination and SARS-CoV-2 infection
Source: Orphanet J Rare Dis. 2023 Mar 10;18:50. doi: 10.1186/s13023-023-02651-3 (PMC9999054; doi:10.1186/s13023-023-02651-3)

**Supplementary Material**

**Figure S1: Demographics for Primary cohort (AOSD)**


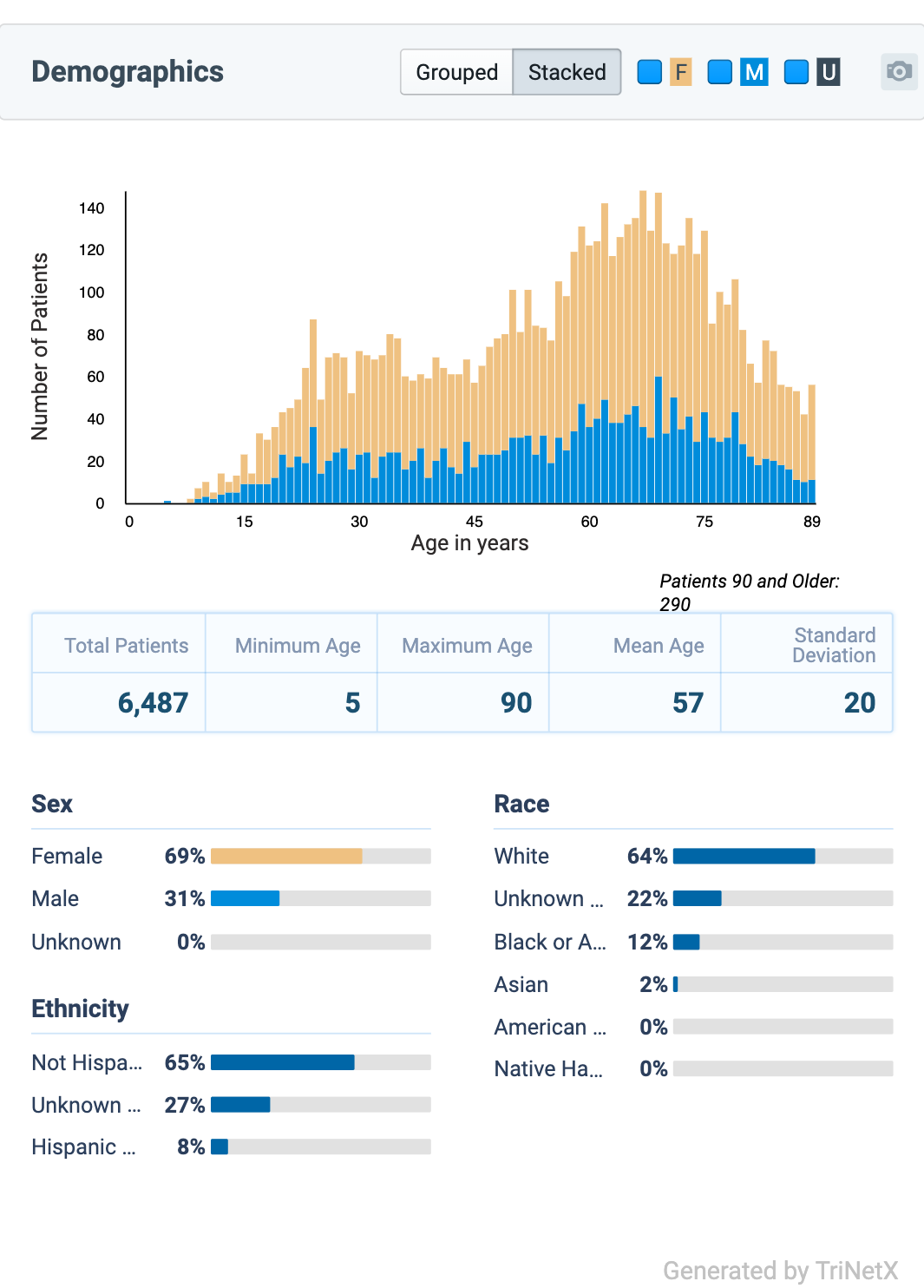


**Figure S2: Demographics for Cov cohort (AOSD + SARS-CoV-2 infection)**


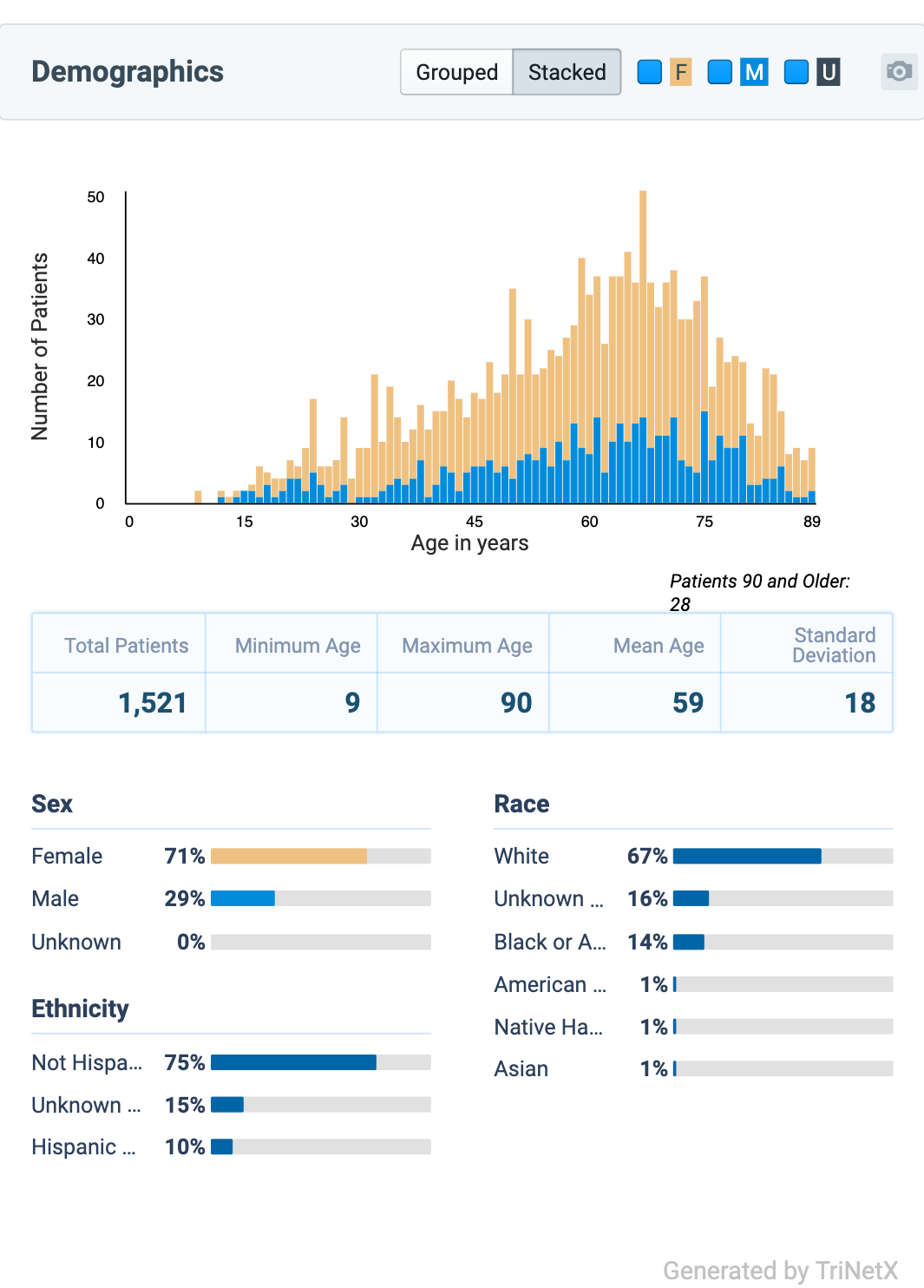


**Figure S3: Demographics for Vac cohort (AOSD + COVID-19 vaccination)**


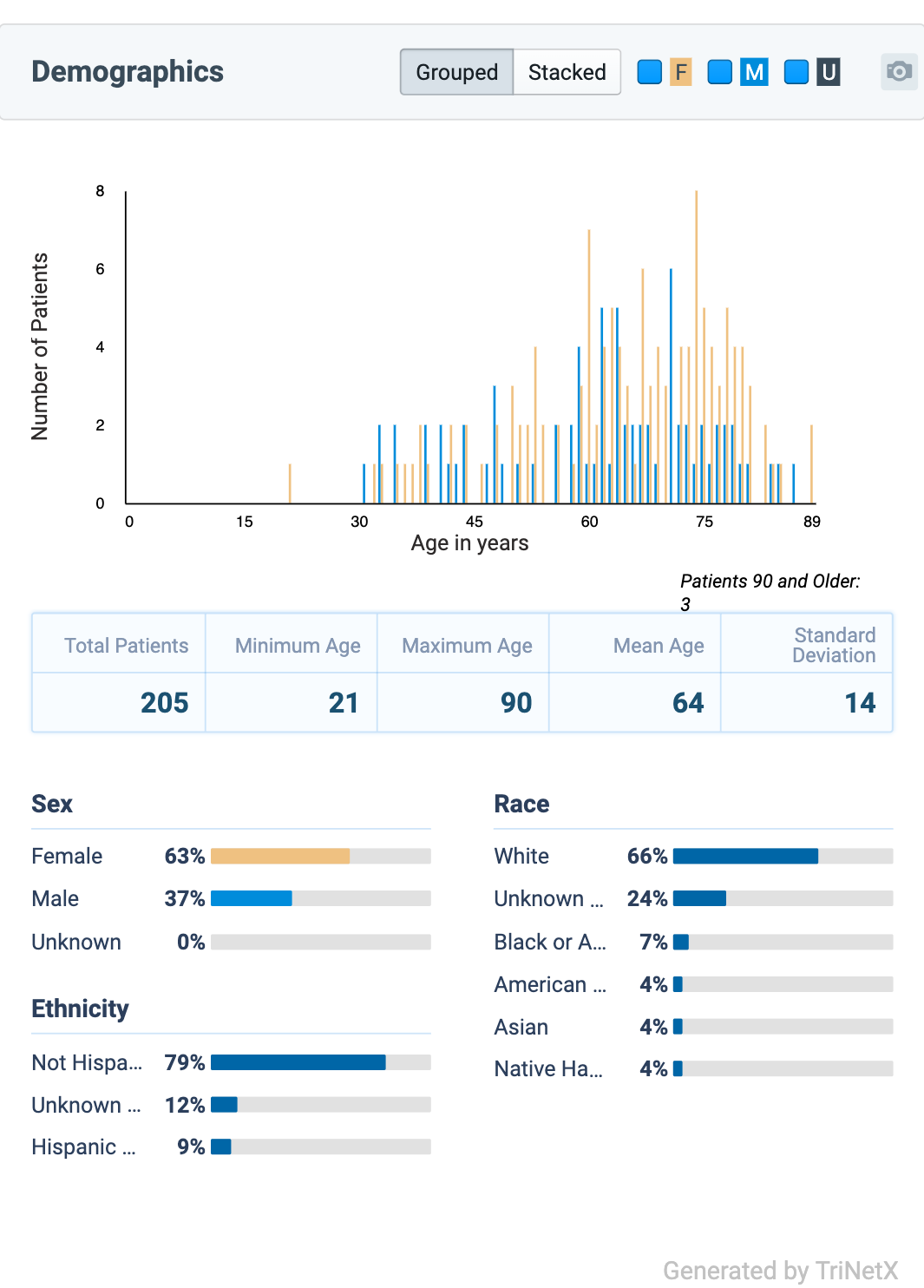


**Figure S4: Demographics for Vac+Cov cohort (AOSD + COVID-19 vaccination + SARS-CoV-2 infection)**


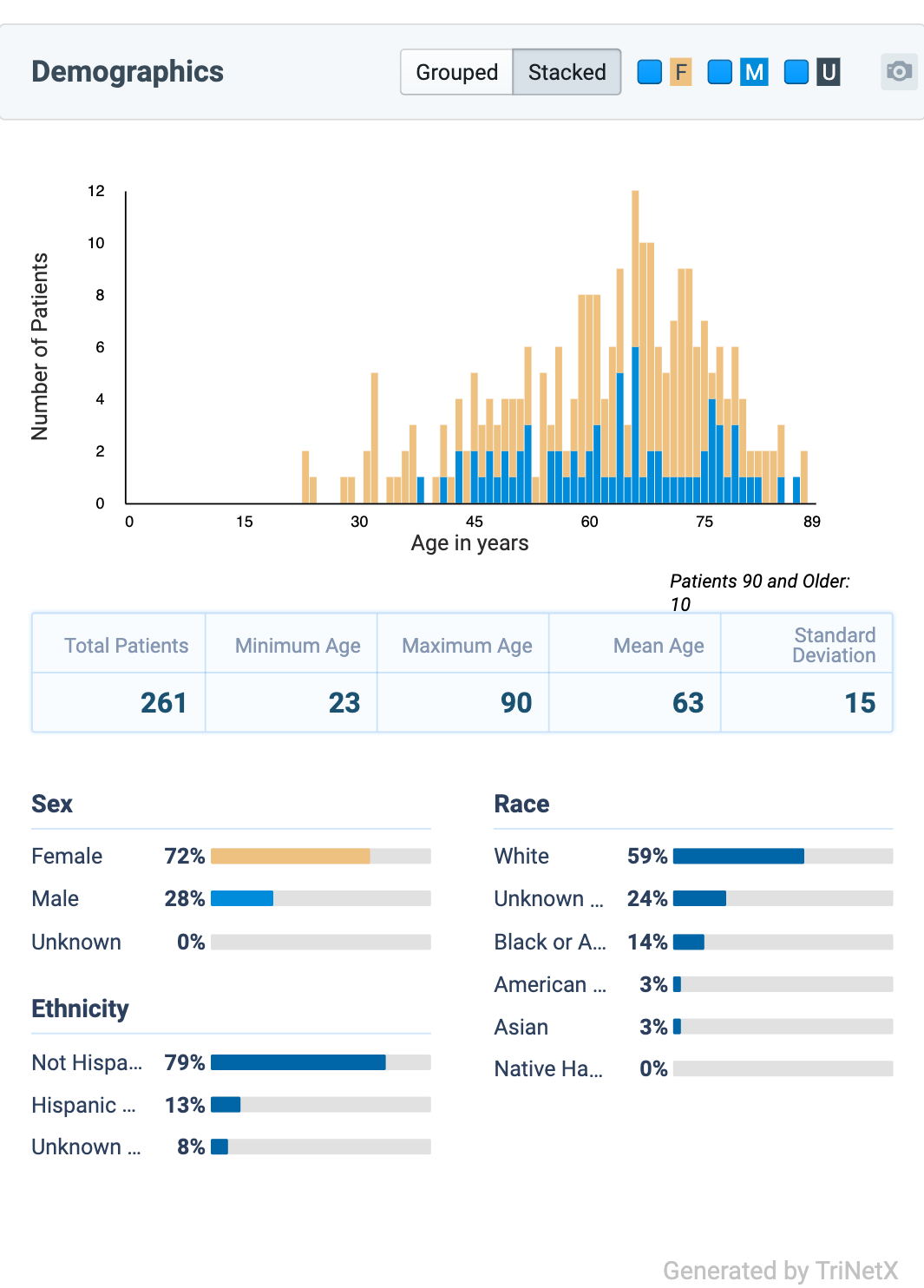


**Figure S5: Modified CONSORT flow chart for the different cohorts.**


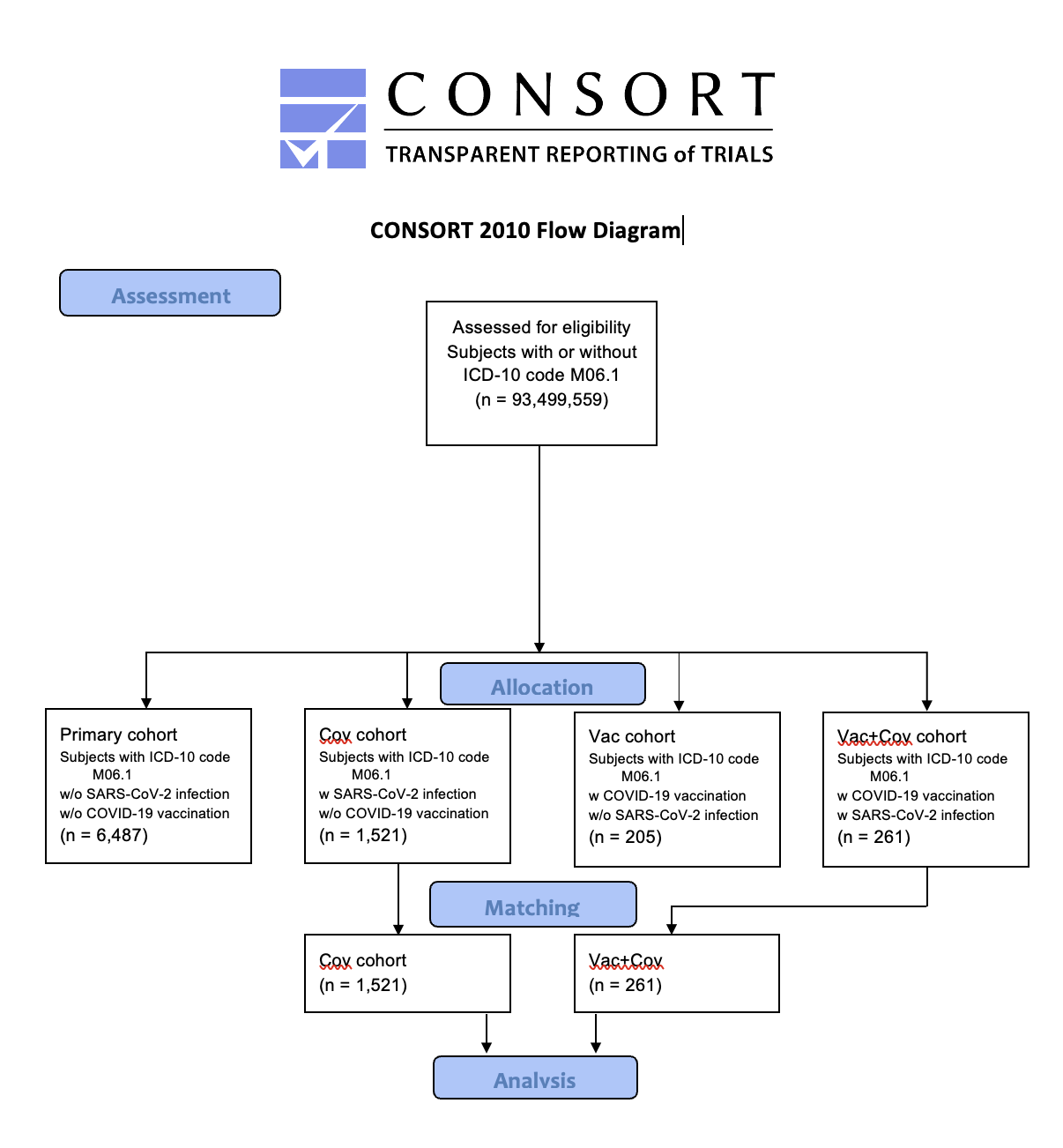

Supplement: Supplementary file 1 — Additional file1. Figure S1: Demographics for Primary cohort (AOSD). Figure S2: Demographics for Cov cohort (AOSD + SARS-CoV-2 infection). Figure S3: Demographics for Vac cohort (AOSD + COVID-19 vaccination). Figure S4: Demographics for Vac+Cov cohort (AOSD + COVID-19 vaccination + SARS-CoV-2 infection). Figure S5: Modified CONSORT flow chart for the different cohorts. [file 13023_2023_2651_MOESM1_ESM.docx]
